# Determinants of patient behavioural loyalty on primary health centres: Evidence from a cross-sectional study in Indonesia

**DOI:** 10.12688/f1000research.110684.2

**Published:** 2022-05-06

**Authors:** Mardaleta Mardaleta, Abdul Rahman Lubis, Yossi Diantimala, Heru Fahlevi

**Affiliations:** 1 Doctoral Program of Management Science on Public Sector Accounting, Faculty of Economics and Business, Universitas Syiah Kuala, Banda Aceh, Aceh, 23111, Indonesia; 2 Doctoral Program of Management Science, Faculty of Economics and Business, Universitas Syiah Kuala, Banda Aceh, Aceh, 23111, Indonesia; 3 Accounting Department, Faculty of Economics and Business, Universitas Syiah Kuala, Banda Aceh, Aceh, 23111, Indonesia

**Keywords:** Service provider, service process, service environment, quality of service, behavioural loyalty, health centre

## Abstract

**Background**: Patients’ loyalty to visit and use the services provided by the primary health centers (PHCs) is an important requirement of a patient referral system in many countries. The aim of this study was to examine the influence of internal service factors (service provider, service process, and service environment) on service quality and behavioural loyalty of patients in Indonesian PHCs.

**Methods**: A cross-sectional study was conducted in 14 districts in Aceh Province, Indonesia between September and December 2020. Data were collected in 102 PHCs that were selected randomly from 137 PHCs that have an Inpatient Unit in the province. A proportional number of patients were recruited from each PHC and 389 patients were included. The demographic data, three components of internal service factors (service provider, service process, and service environment), the service quality and behavioural loyalty were assessed using a validated questionnaire. Hypothesis testing was conducted by using the structural equation model (SEM).

**Results**: Our data suggested that two elements of internal service factors (service provider and service environment) had a positive and significant influence on service quality of the PHCs with p<0.001 and p=0.021, respectively. Service quality had a positive and significant influence of behavioural loyalty of patients to the PHCs (p=0.003). Service quality however did not serve as an intervening variable between internal service factors (service provider, service process, and service environment) and behavioural loyalty of patients, with p=0.091, p=0.230 and p=0.260, respectively.

**Conclusions**: Service provider and service environment are two main factors that influence the service quality and the service quality directly influence the behavioural loyalty on PHC users. Therefore, to increase the patients’ loyalty to use the PHC services, the quality of the services should be improved by levelling up the quality of providers and both physical and social environments in the PHCs.

## Introduction

Patient intention to visit primary health centers (PHCs) is an important requirement of a patient referral system. In many countries, for instance Indonesia, individuals are encouraged to visit their local PHCs first and when it is required, they will be referred to public hospitals. As a gatekeeper to hospitals, facility of PHCs in Indonesia have already improved particularly after the implementation of universal coverage ( Ekawati *et al.*, 2017). However, some studies reported lack of intention to visit and revisit PHCs ( Ulandari & Yudawati, 2019) and dissatisfaction to the PHCs service ( Ekawati *et al*., 2017). Loyal patients that continue to visit the health care provider and follow medication procedures could improve health care services, patient referral system, and patient outcomes ( Zhou *et al.*, 2017). On the contrary, patients reluctant to visit PHCs may lead to unintended impact on the effectiveness of treatment and medication as they do not receive complete and continue medications, and thus, it may lead to low quality of community health.

Previous studies have documented a significant role of service quality on user loyalty. In health care setting, service quality is a crucial factor of an effective health care system ( Mosadeghrad, 2014). Service quality of PHCs is very crucial for a health care system because PHCs are the main entrance and the provider of basic and promotional health care for the population. Failure to achieve expected service quality, means that people may be reluctant to visit or revisit PHCs and would prefer to visit public hospitals directly ( Ulandari & Yudawati, 2019). As a result, the effectiveness of the health care sector would become deteriorated and continuous spikes of patient numbers in public hospitals cannot be avoided.

Defining and measuring health service quality are challenging tasks as service quality is intangible and related to patient perception and expectation ( Mosadeghrad, 2014). From the perspective of patients, service quality is determined and evaluated from their experiences and their interactions with the environment of a hospital or PHC, and interpersonal factors, such as the responsiveness and kindness of staff ( Lam, 1997; Amin & Nasharuddin, 2013). Besides, service quality can potentially lead to the creation of patient behavioural loyalty, which is beneficial for both patient and PHC management. There is a body of literature on predictors of customer perceived service quality. However, similar studies in the context of health services are still scarce ( Rose *et al.*, 2004). Service quality in health care providers can be associated with two primary factors, namely, internal and external factors. Using patients as the respondents, Rose *et al.* (2004) found that interpersonal qualities or human dimension and physical environment quality play a significant role in the hospital service quality of Malaysian hospitals. Mosadeghrad (2014) revealed that the personality of physicians and the patients affect the health care service quality of Iranian hospitals. Meanwhile, Amin & Nasharuddin (2013) investigated the influence of admission, medical service, overall service, discharge, and social responsibility on hospital service quality. They found that the five variables which developed from the service quality (SERVQUAL) model has a significant relationship with hospital service quality.

Although the literature on the impact of internal service factors, for instance, physician characters and hospital environment, regarding service quality is growing, studies on the relationship between service quality and behavioural loyalty are still limited, particularly in the context of primary health care and a developing country ( Murti *et al*., 2013). Zhang & Yang (2018) note that determinants and mechanisms of patient loyalty in the health care industry still remain unexplored.

On the contrary, Aladwan *et al.* (2021) documented a significant impact of service quality on patient loyalty and patient satisfaction in a Jordanian hospital. In addition, Krishna *et al.* (2016) investigated the relationship between health care service quality, patient satisfaction, and behavioural intentions in large hospitals in India. They found that from the five dimensions of SERVQUAL, only empathy affects the behavioural intention of the patients.

As research on the relationship between health care service and behavioural loyalty of patients is still limited, the present study aims to examine the influence of internal service factors (service provider, service process, service environment) on service quality and behavioural loyalty in Indonesian primary health centres. Further, the SERVQUAL dimensions have been validated in the Western world, and there is a possibility that the cultural differences of consumers will affect its applicability, particularly in the context of a public health care system ( Amin & Nasharuddin, 2013).

As an emerging economy, Indonesia has a unique setting for its health care system, that reflects the important role of social insurance and a relatively strict patient referral system ( Fahlevi *et al*., 2021; Handayani *et al.*, 2018). Specifically, Indonesian PHCs are selected as research samples because, firstly, they play a crucial role in the Indonesian health care sector as a primary health care provider ( Rawung & Sholihin, 2017) and, secondly, Indonesia adheres to the principle of tiered and systematic patient referrals, starting from the PHCs, as a first-level health service facility ( Fahlevi, 2016). However, many patients prefer to directly visit public hospitals although Indonesian PHCs are able to deliver treatment for more than 144 types of diagnoses and are mostly free of charges. PHCs are often placed to obtain unnecessary referral permits to hospitals ( Goniwala, 2017). As a result, there has been a surge in patient numbers in Indonesian public hospitals ( Gasim, 2015).

This study contributes to the literature of service quality and behavioural loyalty in health care industries. Prior studies have mostly been conducted in hospital settings in developed countries, for instance the United States (US), the United Kingdom (UK), and Australia ( Pai & Chary, 2011). There are only a few studies that were undertaken in developing and non-Western countries. Moreover, unlike prior studies that were mostly carried out in hospital settings, the present study was conducted in a unique setting, namely PHCs, known as *Puskesmas* in Bahasa Indonesia, as the frontline of the health care sector in most developing countries. Besides, this study tested the mediating role of service quality on the relationship between internal service factors and behavioural loyalty. The role of service quality as an intervening variable of the relations between internal factor services and behavioural loyalty is still unexplored.

## Literature review

Patients develop perceptions during the process of health care delivery and compare them to their expectations ( Wu, 2011). The result of this process is service quality based on patient perspective. Therefore, service quality depends on the nature, context, and scope of the service expected ( Fairchild *et al.*, 1997). Service quality can also be defined as value, excellence, and conformity with the predetermined specifications and requirements of a service ( Grönroos & Ojasalo, 2004; Mosadeghrad, 2014). The dimensions of SERVQUAL that are widely accepted are tangibility, empathy, reliability, responsiveness, and assurance which can be studied in order to understand their impact on service quality ( Zeithaml *et al*., 2002). However, these dimensions need to be validated as culture and social dimensions may different from one country to another.

Previous studies classified quality dimensions into two parts, namely, technical quality and functional quality ( Krishna *et al.*, 2016). The former deals with medication technical accuracy in which the health care providers (doctors, nurses and other supporting medical staff) are the main actors. Technical quality is mirrored by the accuracy of diagnoses and procedures, as well as the effectiveness of medical protocols. Patients have limited knowledge and capacity to measure technical quality. The latter is related with the process of health care delivery. For instance, the interpersonal relationship between doctor and patient, the quality of the hospital environment, and the system. These factors are easily evaluated by patients. Therefore, marketing and business researchers focus more on functional quality.

The internal service factors that potentially contributes to service quality are service provider character, service process, and service environment. A service provider is a person that delivers health care services to patients ( Paulus *et al.*, 2016). Service providers are doctors, nurses, midwives, and other supporting workers in the hospital, or other health care providers. As they are human beings, they have different character and interpersonal skills that contribute to the way they deliver services.

The service process is the capacity of the health care provider in providing health services at the right time, whenever they are needed ( Ferreira & Marques, 2019). The service process requires input, transformation processes, and results ( Okoye & Obeta, 2015), and all are reflected by waiting time, atmosphere in the health care provider, and other dimensions.

The service environment is a condition that meets the convenience of service users such as location, parking, waiting rooms, examination rooms, and cleanliness, and has a variety of media and information centres that can please the service users ( Lovelock & Wirtz, 2016; Meier & Krug, 2009). It also can be in the form of physical facilities such as infrastructure, medical equipment, hygiene of medical staff, and other important health conditions ( Fatima *et al.*, 2018). The service environment is also a medical facility that can attract many service users with high utility ( Meesala & Paul, 2018).

Meanwhile, behavioural loyalty is the result of repeated satisfaction of quality in a service provider ( Fatima *et al.*, 2018). Kumar *et al.* (2006) describes alternative forms of loyalty, namely, behavioural loyalty. Wu (2011) believes that behavioural intention is more suitable for measuring patient loyalty, but the other authors argue that loyalty should be viewed as attitude. Nevertheless, loyalty in every business industry has the same benefits ( Wu, 2011), namely, improving visits and the use of services.

## Hypothesis development

### Service provider and service quality: Service providers affect the service quality of PHCs (Hypothesis 1)

The service quality of the health care provider is a multi-dimensional concept ( Ghahramanian *et al.*, 2017) and providing high-quality service in the health care sector is the key to success in achieving better public health outcomes. From the perspective of patients, service providers can be the most influential factor of perceived service quality. Service providers are doctors, nurses, and other staff in hospitals or other health care organizations, and they interact directly with patients. Their interpersonal skill builds patient perception in the quality of health care and shapes the behavioural loyalty of the patients ( Mosadeghrad, 2014; Fiaz *et al.,* 2018).

The service provider plays a significant role in both technical and functional quality dimensions. The former refers to the technical precision of the medical diagnoses and treatments according to medical professional specifications and standards, while the latter deals with the way patients receive a health care service ( Padma *et al.*, 2010). Physicians are the main actors in diagnosing and deciding on the medical treatment to be delivered to patients and at the same time, they are the dominant person interacting with the patient. As a result, the service provider is among one of the most important determinants of service quality, and both are measured by patients and other stakeholders.

Previous studies have revealed that service provider character and personality affect service quality. For instance, Mosadeghrad (2014) investigated factors that contributed to health care quality in Iranian health care providers. They found that service quality was shaped by the character and personality of patients, doctors, and environmental factors. Moreover, ( Atinga & Baku, 2013) studied determinants of service quality of antenatal care in Ghana. The study uncovered how physician attentiveness contributes to a positive impact on service quality. Based on the above discussion, the first hypothesis proposed is as follow:

### Service process and service quality: Service process affects service quality of PHCs (Hypothesis 2)

The indicators of a good service process can be how fast patients receive the medical treatment, since they register any interactions between patients and staff in the health care facility ( Elleuch, 2008). The service process can also be measured by consistency in terms of quality and standards in health care provision.

A good service process, for example, a shorter waiting list, will improve service quality ( Atella *et al.*, 2019). The service process affects service users, but it depends on several factors such as the nature, character of service users, and waiting times ( Wood *et al.*, 2009). Previous investigators found a relationship between service process and service quality. For instance, Elleuch (2008) studied Japanese patient satisfaction and found that it is determined by process character (patient-doctor interaction) and physical attributes (settings and appearance).

### Service environment and service quality: Service environment affects service quality of PHCs (Hypothesis 3)

The service environment refers to the atmosphere of the health care provider that is created by both the physical environment and the social environment, that shape the patient impression. A good service environment is reflected by a comfortable condition, both outside and inside the building, with a friendly and informative social environment supported by various facilities, and infrastructure, based on policies to improve the quality of PHCs services ( Meier & Krug, 2009).

Moreover, the service environment also relates to social experiences gained by patients during their visits to the health care provider. This includes patient-friendly environment, responsiveness, and interaction between patient and health care providers. In their study in Pakistan, Fatima *et al.* (2018) revealed a positive association between health care service quality aspects, for instance, physical environment and customer-friendly environment, with patient loyalty through patient satisfaction.

### Service quality and behaviour loyalty: Service quality of PHCs affects behavioural loyalty of PHCs users (Hypothesis 4)

In the health care sector, increasing access to hospitals through social insurance may lead to increasing patient interest and concern regarding the quality of health care services ( Fatima *et al.*, 2018). Notwithstanding, the rapid growth of private hospitals and private clinics provides alternatives for patients in selecting which hospital they want to visit. Thus, patient behaviour loyalty has become an important issue, both in a relatively strict referral system or a relatively less strict referral system. In this issue, service quality seems to be a key factor of the loyalty of patients.

Ngoma and Ntale (2019) believe that loyalty encourages users to repeat their use and provide positive feedback to the other members of the public, that would encourage actual and prospective users. Fatima *et al.* (2018) classified two types of loyalty, namely, attitudinal and behavioural loyalty. Attitudinal loyalty refers to loyalty created by distinctive sentiments of customers towards a product or service, while behavioural loyalty can be defined as repeating use of certain products or services from the same providers, expanding the use volume and promoting the product or service among other people ( Fatima *et al.*, 2018). The present study has a focus on behavioural loyalty.

Patients normally use their prior service experience of using health care services to decide whether they would wish to, or not wish to, visit the health care service again. A body of literature has confirmed that the relationship between service quality and behavioural loyalty is emerging. Fatima *et al.* (2018) examines the influence of service quality on patients’ satisfaction and patients’ behavioural loyalty in six private hospitals in Pakistan. Those authors unveiled the fact that better quality of health care services may increase patient satisfaction and loyalty. Ambrose *et al.* (2016) undertook their study in the southern US, and examined the association between service quality and patients’ willingness to recommend a hospital to their relatives and friends. The study revealed that service quality has a positive influence on recommendation behaviour among patients.

The above literature review shows that the relationship between service quality and behavioural loyalty in health care industry is still relatively scanty. Most studies have been conducted in other sectors, for instance in banking industry. Besides, prior studies have been undertaken mostly in developed countries and in a hospital setting. Service quality and behavioural loyalty in the context of PHCs are still uncovered. Unlike hospitals, PHCs have different setting of organisation and limited offered services. For instance, PHCs only provides basic medication for their patients. However, it has an unreplaced role as a gatekeeper to hospital.

**Figure 1.**
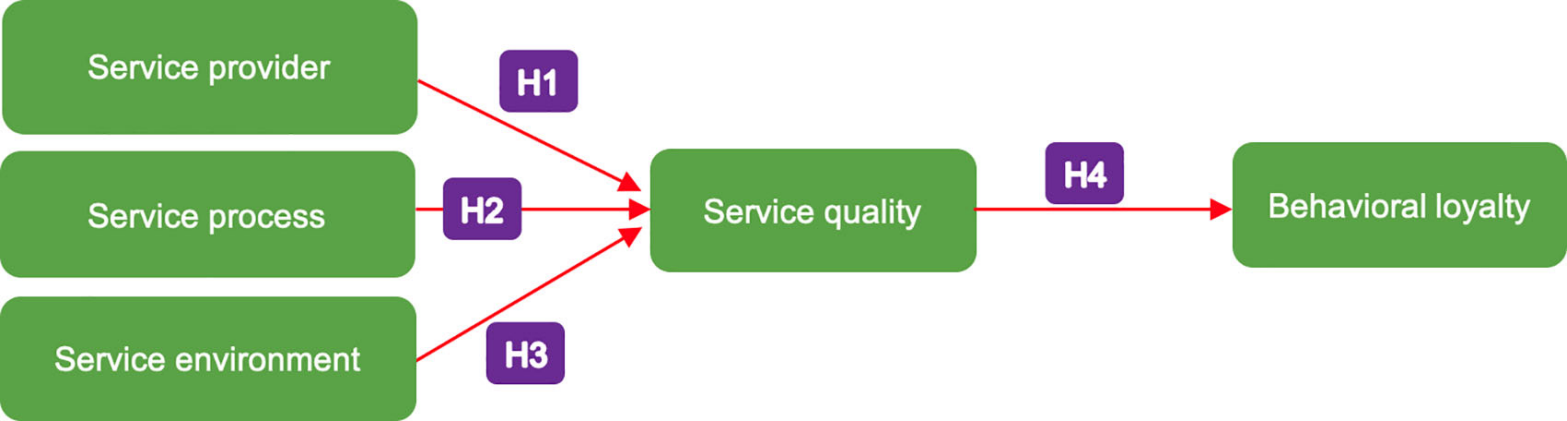
Proposed model of the relationship of service provider, service process, service environment, service quality and behavioural loyalty of patients on primary health centres. H1–H4 indicate the hypotheses that are tested in this study.

Referring to the theoretical framework of the relationship between variables as described previously, the model to be tested in this study is depicted in Figure 1. This research aims to test the aforementioned hypotheses as well as to test the mediating role of service quality on the relationship between internal service factors (service provider, service process and service environment) and behavioural loyalty.

## Methods

### Research design

A cross-sectional study, adhering to the STROBE guidelines ( Mardaleta, 2022b), was conducted in 14 out of 23 districts in Aceh Province, Indonesia to assess the determinants of patient behavioural loyalty on PHCs. The PHC users, patients who received the services from PHCs, were interviewed directly. The questionnaire-assisted interviews were conducted by the authors after receiving the consent from the patients. The data collection was conducted between September and December 2020. Due to the coronavirus disease 2019 (COVID-19) pandemic, we were unable to interview all the patients directly and therefore the questionnaires were provided to the patients and were then collected. The patients were provided with the phone number of the investigator and therefore were able to ask the questions to the investigators during completing the questionnaire.

The protocol of this study was approved by Komisi Program Studi Pendidikan Doktor Ilmu Manajemen, Universitas Syiah Kuala, Banda Aceh, Indonesia (No. B/359/UN11.1.1/DIM/TD.06/2020). The patients provided written consent prior to being included in this study. Due to the COVID-19 pandemic, some patients provided verbal consent only, and the interview was conducted in a social distancing manner. In such conditions, the investigators wrote the notes stating that the patients agreed to participate and signed the consent sheet. Such an approach was approved by the Indonesian ethical committee due to force majeure to avoid the transmission of the virus between patients and investigators.

### Sample and sampling method

Using purposive sampling method, 14 out of 23 districts in Aceh Province were selected based on regionalisation namely, the Central, South, West, and East region. In total, there are 137 PHCs ( *Puskesmas*) that have Inpatient Unit in Aceh Province. The number of PHCs included in this study was determined using the Slovin minimum sample formula: ( $n=\frac{N}{1+N\;{\left(5\%\right)}^{2}}),\;$ where $n=\frac{137}{1+137\;{\left(5\%\right)}^{2}}\;$ = 102 PHCs. Therefore, 102 PHCs were randomly selected using Randomizer, an online randomization tool. Although Slovin formula is a back-of-the-envelope calculation to calculate the number people to be included a study, in this present study the formula was used to calculate the number of PHC which could be represented the sample of the study. The patients who received the services from the selected 102 PHCs, were selected proportionally based on the annual number of inpatient patients of each PHC (ranged between 2–10 patients each PHC).

### Study variables

To assess the factors associated with behavioural loyalty of PHC users, this study assessed two main variables: exogeneous variable (internal service factors) and mediating variable. In this study, exogeneous variables are also called internal service factors since all the variables are part of the PHCs. The exogeneous variable has three main components: service provider, service process, and service environment. Detailed operational definition of each variable are provided in Table 1. In this study, we also examined the role of service quality as an intervening variable of the relationship between internal service factor and behavioural loyalty. Previous studies have shown a relationship between service quality and loyalty. For instance, Wu (2011) found a direct impact of service quality on re-visit intention of patients in Taiwanese hospitals. Krishna *et al.* (2016) demonstrated the indirect effect of one health care service quality, namely, empathy, to behavioural intention. Therefore, the role of service quality to mediate the impact of internal service factors on behavioural loyalty is supported by the previous literature.

**Table 1.** Operational definition of study variables.

Variable	Component	Definition	Indicators	Reference
Exogeneous variable or internal service factors	Service provider	Service providers are doctors, nurses, midwives, and other non-health workers who have social sensitivity, character, and competencies according to the goals, objectives, and standards that have been set by the organization.	1. Social sensitivity 2. Competence (knowledge and expertise) 3. Motivation and satisfaction 4. Professionalism	( Mosadeghrad, 2014; Paulus *et al.*, 2016)
	Service process	Service process refers to how fast and precise are the service provision and standard operating procedures of the organization is implemented.	1. Service speed 2. Service standard 3. Ease of receiving service information	( Atella *et al.*, 2019; Ferreira & Marques, 2019; Meier & Krug, 2009; Okoye & Obeta, 2015; Mosadeghrad, 2014)
	Service environment	Service environment is proxied by comfortable condition, both outside and inside the building, with a friendly and informative social environment supported by various facilities and infrastructure, based on policies to improve the quality of primary health centers services.	1. Physical environment 2. Social environment	( Fornara *et al.*, 2006; Lovelock & Wirtz, 2016; Meier & Krug, 2009)
Mediating variable	Service quality	Quality depends on the nature, context, and scope of the expected service whose indicators can be seen from reliability, responsiveness, assurance, attention, and tangibles, including the dimensions of efficiency.	1. Reliability 2. Responsiveness 3. Assurance 4. Empathy 5. Tangibles	( Parasuraman *et al.*, 2005; Fairchild *et al.*, 1997; Zeithaml *et al*., 2002)
Endogenous variable	Behavioural loyalty	Behavioural loyalty is characterised by frequent use of services and giving positive impressions and reviews It also deals with intention and commitment that affect not only loyalty but also recommends to others to continue treatment and care.	1. Positive impression 2. Directing and suggesting 3. Intention and commitment 4. Treatment and follow-up care	( Ahmed *et al.*, 2017; Fatima *et al.*, 2018)

### Study questionnaire and its assessment

The questionnaire was developed based on previous literature ( Ahmed *et al.*, 2017; Atella *et al.*, 2019; Fairchild *et al.*, 1997; Fatima *et al.*, 2018; Ferreira & Marques, 2019; Fornara *et al.*, 2006; Lovelock & Wirtz, 2016; Meier & Krug, 2009; Mosadeghrad, 2014; Okoye & Obeta, 2015; Parasuraman *et al.*, 2005; Paulus *et al.*, 2016; Zeithaml *et al.*, 2002). The questionnaire consisted of four parts. The first part collected the demographic data while the last three parts assessed the exogeneous, mediating and endogenous variable domain (see the questionnaire in the *Extended data* ( Mardaleta, 2022a)). For exogeneous, mediating and endogenous variables, the possible answers were provided in the Linkert-scale ranged from “Strongly disagree (score 1)” to “Strongly agree (score 5)”. The raw scores were used in the final analysis.

The convergent validity and discriminant validity tests were conducted to ensure the validity of the questionnaire. Based on previous literature, the validity of the questionnaires is confirmed if the loading factor at convergent validity is higher than 0.5 ( Ghozali, 2014). Discriminant validity is verified if the cross loading at the construct is higher than another construct and average variance extracted (AVE) is higher than 0.5 ( Ghozali, 2014). The reliability of the questionnaires is considering good if composite reliability (CR) value is higher than 0.5 and the value of Cronbach’s Alpha higher than 0.6 ( Malhotra & Morris, 2009). Our validity tests showed that all questionnaires’ items were valid as p-value of convergent validity, discriminant validity and AVE were all > 0.50. All of the items were also reliable because the CR value was > 0.70 and Cronbach’s Alpha was > 0.60. Outer loading of each indicator of all variables ranged between 0.703 and 0.913. These indicated that the items within questionnaire are valid and reliable.

### Data analysis and hypothesis testing

The multicollinearity possibility was assessed by using Tolerance dan Variance Inflation Factor (VIF). We found that the VIF value was smaller than 10 indicating no multicollinearity between the domains. To examine the data normality, critical ratio of skewness and kurtosis ± 2.58 were used. Hypothesis testing was carried out by using the structural equation model (SEM). This model was used because this model has several advantages. Firstly, SEM analysis is able to carry out complicated tests of decision-making processes in various public sector management and accounting sciences, and others. Secondly, SEM can be used to address both regressive and dimensional research questions, and it can measure the influence of theoretically existing relationships, including mediating relationships ( Ferdinand, 2014, Holbert & Stephenson, 2003). Thirdly, SEM can analyse down to the level of indicators, or find the root of the problem, because it is not limited to observed variables. Therefore, SEM is the most appropriate method to solve complex and difficult problems, as SEM can distinguish empirical data and latent data that can be defined from the error value and loading factor. Lastly, SEM can perform regression analysis, factor analysis, and path analysis. The confirmatory factor analysis was used to determine the value of the loading factor. The initial measurement model was carried out to examine the goodness of fit value. All analyses were conducted using IBM SPSS Amos version 23.

## Results

### Respondents’ characteristics

During the study, 402 questionnaires were distributed and all of them were returned. However only 389 respondents answered all the questions completely and were included in the final analysis ( Mardaleta, 2022a). The characteristics of the respondents are provided in Table 2. More than half (59%) of the respondents were married and there was an equal gender proportion between male and female (49% vs 51%). Most on the patients were aged between 18 and 54 years old and almost half of them (48%) completed the senior high school. Based on the type of occupation, only 5% of respondents were working as civil servant while the rest were working in non-civil servant sector ( Table 2).

**Table 2.** Respondents’ characteristics (n=389).

Characteristic	Group	Frequency	Percentage
Marital status	Married	228	59%
	Not married	161	41%
Gender	Male	189	49%
	Female	200	51%
Age	Under 18	14	4%
	Between 18 and 29	183	47%
	Between 30 and 54	171	44%
	More than 55	21	5%
Educational attainment	Primary school	36	9%
	Junior high school	61	16%
	Senior high school	188	48%
	Vocational/diploma program	23	6%
	Undergraduate	78	20%
	Postgraduate	2	1%
	Non-formal education	1	0%
Occupation	Civil servant	19	5%
	Not-civil servant	370	95%

### SEM results

Our assessment of criteria measurement of SEM indicated that the data were normal and there were no outliers indicating the data could be used to test our SEM structural model and the hypotheses. An evaluation of the goodness of fit criteria of a model with several index suitability criteria and a cut off value was conducted in order to ensure whether a model can be accepted or rejected. Our SEM model had a p-value > 0.05, chi-square fit statistics/degree of freedom was less than 2 goodness-of-fit index (GFI), root mean square error of approximation < 0.08, Tucker Lewis index and normed fit index were both > 0.90, parsimonious goodness-of-fit index <1.0 and GFI > 0.90. These suggested that our SEM model was acceptable.

The SEM test was assessed the influence between variables; if the variable has a probability value p < 0.05, the hypothesis is accepted. The results of the SEM analysis are provided in Table 3. It can be clearly seen that not all of the hypotheses are supported by the study. Two components of internal service factors, service provider and service environment, had a positive and significant influence on service quality of the PHCs with p < 0.001 and p = 0.021, respectively. The service process has a positive, but insignificant, impact on service quality. Our data also suggested that service quality, indeed, was a determinant of behavioural loyalty of patients to the PHCs (p = 0.003) ( Table 3).

**Table 3.** Direct effect and regression weight of structural equation model (SEM).

Relationship	Estimate	p-value	Conclusion
Service provider → Service quality	0.544	< 0.001	Hypothesis is supported
Service process → Service quality	0.226	0.070	Hypothesis is not supported
Service environment → Service quality	0.256	0.021	Hypothesis is supported
Service quality → Behavioural loyalty	1.095	0.003	Hypothesis is supported

We also assessed the intervening role of service quality. Here, we assessed its role as mediator variable that influences the relationship between the independent variable (internal service factors) and the dependent variable (behavioural loyalty). Our data suggested that service quality did not serve as an intervening variable between internal service factors (service provider, service process, and service environment) and behavioural loyalty of the patients, with p = 0.091, p = 0.230 and 0.260, respectively ( Table 4).

**Table 4.** Test result of intervening role of service quality.

Relationship	Estimate	p-value
Service provider → Service quality → Behavioural loyalty	0.595	0.091
Service process → Service quality → Behavioural loyalty	0.248	0.230
Service environment → Service quality → Behavioural loyalty	0.280	0.260

## Discussion

This study confirms the influence of two internal service factors (service provider and service environment) on service quality. This is consistent with the study conducted in Iranian hospitals ( Mosadeghrad, 2014). Those authors found that service quality was shaped by the character and personality of doctors (service provider) as well as environmental factors, for instance, resources and facilities owned by the hospital. It is also consistent with the findings of Atinga & Baku (2013), which revealed that physician attentiveness has a significant and positive impact on service quality. However, the present study does not provide empirical proof on the relationship between service process and service quality of PHCs. The dimensions of service process are service speed and service standard. Thus, this finding is not in line with Elleuch's (2008) in the Japanese hospital setting, and it was found that service quality is determined by process character (patient-doctor interaction) and physical attributes (setting and appearance).

Furthermore, the result of this study confirms a positive and significant impact of quality service on patient behavioural loyalty. This is consistent with prior studies, for instance, Fatima *et al.* (2018) in Pakistan and Rehman (2012) in Pakistan, United Arab Emirates, and the UK, Aladwan *et al.* (2021) in the Jordanian hospital setting, and Krishna *et al.* (2016) in India. Fatima *et al.* (2018) found that service quality, reflected by costing, process quality, interaction, and environment quality, affected patient loyalty. Rehman (2012) revealed that service quality of health care service was shaped by patient loyalty, which was mediated through patient satisfaction. In their research, service quality was proxied by privacy and safety, patient-friendly environment, responsiveness, physical environment, and communication.

However, the present study does not confirm the relationship between internal service factors and patient behaviour loyalty through service quality. Thus, the results are not in line with some previous research, for example, Lee (2021) found that the service provider with good communication skills has a positive effect on the cognitive trust of service users that leads to patient loyalty. Moreover, this research finding is also not consistent with research from other sector settings. For instance, Boonlertvanich (2019) studied the impact of customer-perceived service quality on the behavioural loyalty of customers of a large commercial bank in Thailand. This study confirms an intervening role of satisfaction and trust in the relationship between service quality and behavioural loyalty.

The present study suggests that management of PHCs should focus on the improvement of internal service factors, particularly service provider and service environment, as these factors contribute positively to service quality. In doing so, management can ensure that doctors, nurses, and other supporting staff are having good interpersonal skills, motivation, and professionalism in order to provide the best health service to patients. PHCs management should focus on recruiting good personality of service providers. Moreover, PHCs need to maintain both a physical and social environment that shape good perceptions of patients on the service provided by the PHCs. PHCs should invest in improving both medical and supporting facilities, as well as creating a caring social environment. Finally, this study confirms the influence of service quality in behavioural loyalty of patients. Thus, each PHC needs to improve service quality to attract more patients and motivate existing patient to re-visit the PHC. As a result of improved patient loyalty, patients will follow the referral system properly and, thus, public hospitals in Indonesia can have ideal patient numbers. Behavioural loyalty is crucial in the health system because it could represent trust in health care providers and recommendations they provide, willingness to engage within the system and adherence to treatments. In addition, in the context of COVID-19 pandemic the loyalty in the health system might crucial to increase the trust to health care providers that could improve the acceptance of COVID-19 vaccine which is still a main problem in many countries ( Hassan *et al.*, 2021; Rosiello *et al.*, 2021).

### Study limitation

This study is not free from limitations. Firstly, only PHCs in Aceh province were included in this study. Therefore, the generalisation of results is limited. Secondly, this study did not take into account patient characteristics as independent variables that may contribute to the different perception of service quality. Therefore, this study suggests a wider research scope for further research. Qualitative-based research is also suggested to explore patient perception in service quality, particularly in the current pandemic situation. Thirdly, it is suggested for further study to compare determinants of service quality between PHCs and private clinics, to gain a more comprehensive understanding of service quality from the perspective of health care users. Lastly, since the COVID-19 pandemic, we were unable to interview all the patients directly and some of the data were collected through the self-administered questionnaire. Therefore this might could cause bias.

## Conclusions

In a health care system where competition among providers is limited and the patient referral system is a crucial component, patient loyalty is very important. Indonesian public hospitals have been mostly over capacity as the referral system is not working properly. One of the reasons is the service quality of PHCs as the main entrance of the patient referral system. This study found that behavioural loyalty of PHCs patients is determined by quality service, while quality service is shaped by internal service factors, namely, service provider and service environment. Thus, the findings of this study can be used to improve the service quality and behavioural loyalty of the patients and, hence, it will lead to the decline in the number of hospital patient visits.

## Data availability

### Underlying data

Figshare: ‘Determinants of patient behavioural loyalty on primary health centres: evidence from Indonesia’, https://doi.org/10.6084/m9.figshare.19326731 ( Mardaleta, 2022a).

This project contains the following underlying data:

• R_Master Data.xlsx [Table containing the raw data of the study].

### Extended data

Figshare: ‘Determinants of patient behavioural loyalty on primary health centres: evidence from Indonesia’, https://doi.org/10.6084/m9.figshare.19326731 ( Mardaleta, 2022a).

This project contains the following extended data:

• Study Quesionnaire.pdf [Questionnaire used to collect the data during the study]

## Reporting guidelines

Figshare: STROBE checklist for “Determinants of patient behavioural loyalty on primary health centres: evidence from Indonesia”, https://doi.org/10.6084/m9.figshare.19326845 ( Mardaleta, 2022b).

Data are available under the terms of the Creative Commons Attribution 4.0 International license (CC-BY 4.0).
