# Supplementary material for: Determinants of patient behavioural loyalty on primary health centres: Evidence from a cross-sectional study in Indonesia
Source: F1000Res. 2022 May 6;11:440. Originally published 2022 Apr 20. [Version 2] doi: 10.12688/f1000research.110684.2 (PMC9111361; doi:10.12688/f1000research.110684.2)
Supplement: Peer review history

# Peer review: Reviewer response for version 2

**DOI:** 10.5256/f1000research.133744.r136896

**Published:** 2022-05-16

**Authors:** Morteza Arab-Zozani

**Affiliations:** 1 Social Determinants of Health Research Center, Birjand University of Medical Sciences, Birjand, Iran

The manuscript is acceptable in this matter.

 Cheers.

Is the work clearly and accurately presented and does it cite the current literature?

Yes

If applicable, is the statistical analysis and its interpretation appropriate?

Yes

Are all the source data underlying the results available to ensure full reproducibility?

Yes

Is the study design appropriate and is the work technically sound?

Yes

Are the conclusions drawn adequately supported by the results?

Yes

Are sufficient details of methods and analysis provided to allow replication by others?

Partly

Reviewer Expertise:

Health policy

I confirm that I have read this submission and believe that I have an appropriate level of expertise to confirm that it is of an acceptable scientific standard.

# Peer review: Reviewer response for version 2

**DOI:** 10.5256/f1000research.133744.r136895

**Published:** 2022-05-09

**Authors:** Abram L. Wagner

**Affiliations:** 1 Department of Epidemiology, University of Michigan, Ann Arbor, MI, USA

No further comments

 (small typo in "Data analysis and hypothesis testing - should be Tolerance and Variance Inflation Factor)

Is the work clearly and accurately presented and does it cite the current literature?

Yes

If applicable, is the statistical analysis and its interpretation appropriate?

Yes

Are all the source data underlying the results available to ensure full reproducibility?

Yes

Is the study design appropriate and is the work technically sound?

Yes

Are the conclusions drawn adequately supported by the results?

Yes

Are sufficient details of methods and analysis provided to allow replication by others?

Yes

Reviewer Expertise:

Epidemiology, public health, vaccination services

I confirm that I have read this submission and believe that I have an appropriate level of expertise to confirm that it is of an acceptable scientific standard.

# Peer review: Reviewer response for version 1

**DOI:** 10.5256/f1000research.122313.r135573

**Published:** 2022-05-03

**Authors:** Morteza Arab-Zozani

**Affiliations:** 1 Social Determinants of Health Research Center, Birjand University of Medical Sciences, Birjand, Iran

Dear respectable authors,

 Thank you for considering a great area of research related to determinants of patient behavioral loyalty on primary health centers in Indonesia. The focus of this cross-sectional study is investigating the effects of internal service factors on service quality and behavioral loyalty of patients in PHCs in Indonesia. Based on the results, the service provider and service environment are two main factors that influence the service quality, and service quality directly influences the behavioral loyalty of PHC users. Your manuscript is well-written but needs some minor revisions as follows:

- Please include "internal service factors" and "service quality" in your title to make it clearer and more complete.
- Abstract: Please add the sample size in the methods section. It is not clear enough to me.
- What is the basis for developing the hypotheses of this study? Give a brief explanation.
- How is the informed consent form obtained from the patients?
- Are the samples selected in equal proportions from all regions? Otherwise, there is a possibility of sampling error/selection bias.
- Please remove the lines 1- 5 at the start of the results section. These details are not related to the results. Please add these details in the methods section and also in the limitations of the study.

Cheers.

Is the work clearly and accurately presented and does it cite the current literature?

Yes

If applicable, is the statistical analysis and its interpretation appropriate?

Yes

Are all the source data underlying the results available to ensure full reproducibility?

Yes

Is the study design appropriate and is the work technically sound?

Yes

Are the conclusions drawn adequately supported by the results?

Yes

Are sufficient details of methods and analysis provided to allow replication by others?

Partly

Reviewer Expertise:

Health policy

I confirm that I have read this submission and believe that I have an appropriate level of expertise to confirm that it is of an acceptable scientific standard, however I have significant reservations, as outlined above.

# Response

**Published:** 2022-05-04

**Authors:** Heru Fahlevi

**Affiliations:** Universitas Syiah Kuala, Indonesia

- We would like to thank you for the suggestion for the change of the title but we prefer our current title since this would be clearer for the readers rather than "internal service factors" which might less common. 
- We have provided the information on the sampling of the respondents and the sample size used in the present study in the abstract. 
- Thank you for the suggestion. We have provided an explanation of how each hypothesis was developed. Please read the text under the subheading of each hypothesis where the basis for developing the hypothesis is provided. 
- We have provided the information on how the informed consent was obtained. It could be seen under the Research design. We wrote: “The questionnaire-assisted interviews were conducted by the authors after receiving the consent from the patients. …The patients provided written consent prior to being included in this study. Due to the coronavirus disease 2019 (COVID-19) pandemic, some patients provided verbal consent only, and the interview was conducted in a social distancing manner. In such conditions, the investigators wrote the notes stating that the patients agreed to participate and signed the consent sheet. Such an approach was approved by the Indonesian ethical committee due to force majeure to avoid the transmission of the virus between patients and investigators."
- Thank you for your suggestion. We have removed some texts about the issue that we faced during the data collection due to the COVID-19 pandemic and moved them to methods and limitations.

# Peer review: Reviewer response for version 1

**DOI:** 10.5256/f1000research.122313.r135575

**Published:** 2022-04-26

**Authors:** Abram L. Wagner

**Affiliations:** 1 Department of Epidemiology, University of Michigan, Ann Arbor, MI, USA

The relationship between patients and health care workers is key to working in medicine and public health. Physicians and other health care workers provide direct clinical care and make recommendations about behavioral changes. Within Indonesia, health care workers within primary health centers, or Puskesmas, are also available to refer patients to specialists, within the Indonesian system of health care coverage.

 How the patient-physician relationship operates is key. A stable relationship can lead to better and more sustained health outcomes in the individual and community. The study by Mardaleta *et al*. examines this relationship within the dimension of loyalty. They developed a model of how service providers, service processes, and service environments can lead to behavioral loyalty through the intermediate variable of service quality.

 Overall, this was a well-written and well-analyzed study. My one major contention that I would want the authors to respond to would be about treating health care as a business. I think there are certain analogies between health care and business, so discussing loyalty is relevant. However, health care isn't a business in the sense that patients are not consumers exactly. Health care providers have larger ethical and professional obligations beyond a typical commercial business. This doesn't negate the analysis, but the introduction or discussion could contextualize what is or isn't transferable from businesses. For example, there is literature on how you can be concerned about providing good patient-centered care but also not treat patients as consumers (e.g. Gusmano *et al*., 2019 ^1^).

 The study sampling frame relied on primary health centers. I assume patterns of loyalty might differ between those who do or do not regularly attend clinics. This could be a limitation.

 Minor issues:

- I would remove the sentence, " Supriyanto *et al.* (2021) studied the influence of service quality on bank customer loyalty in Indonesia. They found that service quality had no significant effects on customer loyalty." - I understand the reason for it, but I think health care is generally different enough from other industries that making cross-industry comparisons could be problematic.
- Likewise, I would delete, "The service process can be defined as the nature and characteristic of the process of delivering service." - it's a bit of a tautology.
- I believe Slovin's minimum sample formula is sort of a back-of-the-envelope calculation for figuring out how many people to sample into a study, whereas you are using it to sample the cluster. I would just state that explicitly.
- You mention that you selected the number of inpatient patients in each Puskesmas proportionally - can you provide a range in the methods or results?
- When you mention SEM analyses, you state, "Thirdly, SEM can analyse down to the level of indicators, or find the root of the problem, because it is not limited to latent variables." - I believe here you mean "not limited to observed variables" because isn't the benefit of SEM that you can measure latent constructs?
- Could you better explain the importance of behavioral loyalty? I see that it could represent trust in health care providers and recommendations they provide, willingness to engage within the system, adherence to treatments or willingness to get screened/get vaccinated, etc.

Is the work clearly and accurately presented and does it cite the current literature?

Yes

If applicable, is the statistical analysis and its interpretation appropriate?

Yes

Are all the source data underlying the results available to ensure full reproducibility?

Yes

Is the study design appropriate and is the work technically sound?

Yes

Are the conclusions drawn adequately supported by the results?

Yes

Are sufficient details of methods and analysis provided to allow replication by others?

Yes

Reviewer Expertise:

Epidemiology, public health, vaccination services

I confirm that I have read this submission and believe that I have an appropriate level of expertise to confirm that it is of an acceptable scientific standard.

## References

[1] : Patient-Centered Care, Yes; Patients As Consumers, No. *Health Aff (Millwood)* .38(3) : 10.1377/hlthaff.2018.05019 368-373 10.1377/hlthaff.2018.05019 30830817

# Response

**Published:** 2022-05-04

**Authors:** Heru Fahlevi

**Affiliations:** Universitas Syiah Kuala, Indonesia

Thank you for reviewing our manuscript. We believe the comments and suggestions improve the quality of our current manuscript 

- We have removed the sentence: “Supriyanto *et al.* (2021) studied the influence of service quality on bank customer loyalty in Indonesia. They found that service quality had no significant effects on customer loyalty”.
- We have removed the sentence: “The service process can be defined as the nature and characteristic of the process of delivering service” from the literature review.
- Thank you for your suggestion. We have provided a brief explanation of the use of Slovin's formula. 
- The range of the included patients from each PHC has been added in the “Sample and sampling method” section.
- Thank you for catching this error. We have revised this. We have corrected an error on the third advantage of the SEM analysis.
- We have provided more explanation of the important of the behavioral loyalty at the end of the discussion including its importance in the context of the COVID-19 pandemic.
